# Gabapentin Does Not Appear to Improve Postoperative Pain and Sleep Patterns in Patients Who Concomitantly Receive Regional Anesthesia for Lower Extremity Orthopedic Surgery: A Randomized Control Trial

**DOI:** 10.1155/2017/2310382

**Published:** 2017-02-27

**Authors:** Jean Daniel Eloy, Christy Anthony, Shawn Amin, Moorice Caparó, Mark C. Reilly, Steven Shulman

**Affiliations:** ^1^Department of Anesthesiology and Perioperative Medicine, Department of Acute Pain, Rutgers New Jersey Medical School, 185 South Orange Avenue, MSB E-538, Newark, NJ 07101-1709, USA; ^2^Department of Orthopedic Surgery, Rutgers New Jersey Medical School, 185 South Orange Avenue, Newark, NJ 07101-1709, USA

## Abstract

In recent years, gabapentin has gained popularity as an adjuvant therapy for the treatment of postoperative pain. Numerous studies have shown a decrease in pain score, even with immediate postoperative activity, which is significant for early post-op ambulation and regaining functionality sooner. However, studies have been in conclusive in patients undergoing lower extremity orthopedic surgery. For this reason, we hoped to study the effect of gabapentin on postoperative pain in patients undergoing total knee arthroplasty, total hip arthroplasty, or a hip fracture repair. This was done in the setting of ensuring adequate postoperative analgesia with regional blocks and opioid PCA, as is protocol at our institution. Given the sedative effects of gabapentin and the potential for improving postoperative sleep patterns, we also studied the drug's effect on this aspect of our patient's postoperative course. We utilized the Pittsburg Sleep Quality Index and Visual Analog Scale for pain to obtain a more objective standardized score amongst our study population. Our results indicate that gabapentin does not offer any additional relief in pain or improve sleep habits in patients who have received either a femoral or lumbar plexus block for lower extremity orthopedic surgery. This trial is registered with NCT01546857.

## 1. Introduction

Gabapentin is a structural analog of the neurotransmitter GABA that has been used to treat epilepsy and neuropathic pain [1]. Gabapentin, despite being structurally similar to GABA, does not have a direct or indirect action on the GABA receptors. Instead, it acts on the *α*2*δ* subunit of voltage gated calcium channels within the CNS and modulates the release of neurotransmitters [2]. In recent years, gabapentin has been used as an adjunct for the treatment of acute postsurgical pain [3] with meta-analyses showing that it significantly reduces postoperative opioid use as well as pain scores [4–6]. A recent meta-analysis showed that perioperative gabapentin reduced opioid consumption and pain scores during the first 24 hours after surgery [7]. The same meta-analysis showed that gabapentin reduced preoperative anxiety, postoperative nausea, vomiting, and pruritus. Recent studies have also supported the use of gabapentin as an adjuvant agent in postoperative pain management [8] in different patient population and after a variety of different surgical procedure. For example, studies have suggested decreased pain with activity in postmastectomy patients [9], and Gilron et al. showed a combination of gabapentin and rofecoxib was superior in relieving pain after hysterectomy than each agent alone [10]. Later, Gilron et al. evaluated the effect of adding meloxicam to gabapentin in patients undergoing outpatient laparoscopic surgery, but data suggested that this provided little additional benefit. Other studies by Clarke et al. demonstrated the decrease in the use of opioids in patients who received preoperative and postoperative gabapentin after total knee arthroplasty [3]. They also noted that optimum timing and dosage of the gabapentin needed to be further elucidated. However, in the same year, Clarke et al. also reported that gabapentin provided no benefit after total hip arthroplasty when a robust multimodal analgesic regimen was combined with spinal anesthesia [11].

One of the side effects of gabapentin includes sleepiness, which may be beneficial to surgical patients in the immediate postoperative period. In a meta-analysis, Peng showed a 35% decrease in total opioid consumption in the first 24 hours postoperatively with the use of gabapentin [5]. Despite the myriad of literature on gabapentin and postoperative pain, little is known about the use of perioperative gabapentin and its effect on sleep quality. While a recent investigation did not find a direct correlation between sleep disturbances and postoperative pain, a questionnaire study found that the most common reason for nighttime awakenings was pain and that analgesia was the most helpful intervention [12]. In healthy subjects, gabapentin has been shown to increase slow wave sleep (SWS), maintain a stable REM, and reduce arousals, awakenings, and stage shifts—all of which are features of sleep fragmentation [13]. Sleep, however, deviates from the normal sleeping pattern in the postoperative patient. Total sleep time, proportion of REM sleep, and proportion of slow wave sleep (SWS) are all reduced. Postoperatively, pain tends to be highly fragmented with multiple spontaneous awakenings and movement arousals. Most of these changes occur during postoperative days 1 and 2; however, patients also incur a REM sleep rebound in days 3 and 4 that can extend up to a week [14]. Studies have demonstrated that these postoperative sleep disturbances, notably the prolonged REM sleep rebound, may contribute to the development of altered mental function [15], postoperative episodic hypoxemia [16], and hemodynamic instability [17]. A recent study even found that postoperative sleep disruptions independently predicted functional limitations three months following surgery in patients who underwent total knee replacement [18]. The pathogenesis of these sleep changes appears to be strongly correlated with the magnitude of the surgery as opposed to the type of anesthesia used. Many specific mediators of surgical stress response have been indicated, including catecholamines, cortisol, and IL-1. However, as REM sleep is controlled by many regions of the brain, disturbances may be due to a global excitatory CNS effect.

The primary purpose of this randomized, double-blinded, placebo-controlled study was to determine whether, in the context of a lumbar plexus or femoral nerve block (depending on the surgery), a single dose of gabapentin improves self-reported measures of a patient's sleep quality in the form of a modified version of the Pittsburg Sleep Quality Index (PSQI), a subjective sleep scale. The secondary endpoint examined whether the addition of gabapentin improves postoperative pain, assessed on both postoperative days one and two.

## 2. Materials and Methods

### 2.1. Patient Sample and Recruitment Procedures

This study was submitted to approval by the IRB and, once obtained, all patients were provided with informed written consent in order to participate in the study. The patients were between 18 and 70 years old undergoing either a total knee arthroplasty, total hip arthroplasty, or a hip fracture repair. The patients selected had an ASA score between I and III. Patients were not eligible to participate in the study if they met any of the following criteria: pregnancy or breast feeding, allergy to any of the drugs to be used in the study or to aspirin and other NSAIDs (such as ibuprofen), a history of a sleep disorder (such as obstructive sleep apnea or daytime somnolence), a history of taking chronic narcotic pain medications or gabapentin, a history of rheumatoid arthritis, a psychiatric disorder, diabetes with nephropathy, a history of alcohol or illicit drug abuse, intrinsic hepatic or renal disease, the inability or unwillingness to use patient-controlled analgesia, inability to meet extubation criteria in the operating room, a history of asthma, a history of stroke or heart attack or thrombotic event within the past 3 months, lactose intolerance, or a history of cardiac surgery.

Once confirmed to be eligible, the patients were recruited for the study. Demographic information was obtained in a detailed preoperative interview the morning of the surgery. Furthermore, a preoperative sleep history was obtained by using a modified version of the Pittsburgh Sleep Quality Index (PSQI), described below. Out of the 50 intended subjects, we were able to enroll only 29 patients, which were randomized into two groups: the placebo group and the gabapentin group.

### 2.2. Drug Preparation, Dispensing, and Randomization

Both the gabapentin and placebo tablets were formulated and encapsulated by the University Hospital research pharmacist. The placebo capsules were composed of a mixture of lactose and granulose. The randomization protocol was completed solely by the research pharmacist, who was not involved in the care of the patients.

### 2.3. Pre-, Intra-, and Postoperative Anesthesia Care

Prior to surgery, all patients were given midazolam 1–3 mg intravenously to achieve anxiolysis. The patients also received a lumbar plexus block or a femoral nerve block, depending on the surgery performed, as this is the current standard of care for orthopedic patients at University Hospital in Newark. All patients received celecoxib 200 mg orally twice daily for three postoperative days. In the operating room, standard general anesthesia technique or neuraxial anesthesia was utilized. Upon extubation, the patients were transferred to the postanesthesia care unit (PACU), where baseline pain and sedation scores were obtained using the Visual Acuity Scale (VAS), the Modified Wilson Sedation Scale (WSS), and Ramsay Sedation Scale (RSS), respectively. All pain scores were assessed with subjects in the resting position. A continuous infusion of bupivacaine 0.125 mg/L was started at a rate of 10 mL/hr and continued to postoperative day 2. An IV PCA hydromorphone pump was initiated and set to deliver a 0.2 mg bolus per demand with a 5-minute lockout period without a basal infusion. If hydromorphone was unavailable PC, Morphine Sulfate was utilized and set to deliver a 1 mg bolus per demand with a 5-minute lockout. At the time of data analysis, for the subjects who received PCA Morphine, their dosages were converted to the equivalent dosage of hydromorphone. All patients were instructed to maintain their VAS pain score at less than 4 out of 10. If the VAS pain score was 5 or greater at rest on two consecutive pain assessments, the dose of intravenous PCA hydromorphone was increased to deliver a 0.3 mg bolus per demand and the PCA Morphine was increased to deliver a 1.5 mg bolus per demand.

In addition to this, a single 400 mg dose of gabapentin was given at 9 pm of the procedure day and another dose was given at 9 pm on postoperative day (POD) 1. All teams involved in the care of the patient were aware not to order any additional sleep adjuvants. The following mornings, on POD1 and POD2, a questionnaire addressing the quality of sleep, hours of sleep, number of awakenings throughout the night, and contributing reasons for these awakenings (pain, noise, urination, temperature discomfort, positional discomfort, for nursing care, or other reasons) was given. Patients were also assessed for pain, sedation, and the incidence of any side effects, including nausea, vomiting, dizziness, and pruritus. In addition, all patients began an as-tolerated weight-bearing rehabilitation program for range of motion, strengthening, balance, and ambulation beginning the first day after surgery. Success in completion of the physical therapy goals of being out of bed in a chair by post-op day 1 and ambulating by post-op day 2 was determined for each group. No functionality testing was done in this study since a previous study was unable to show an association between gabapentin and improvement in function after TKA [19].

### 2.4. Questionnaire

The Pittsburg Sleep Quality Index (PSQI) [20, 21] is a self-rating questionnaire used to measure the quality and other parameters of sleep in adults. The PSQI is comprised of seven categories which are graded and equally weighed on a scale from “0” to “3” with the value of “3” being the most negative endpoint. The categories are subjective sleep quality, sleep latency, sleep duration, sleep efficiency, sleep disturbances, use of sleep medication, and daytime dysfunction over the last month. The seven components are added up to a global score which ranges between “0” and “21” with higher scores indicating worse sleep quality. Studies have implemented the PSQI to assess the relationship between sleep quality and postoperative pain at 1 and 6 months following TKA [18, 22].

We also used the Visual Analog Scale for pain (VAS) as a secondary outcome. The VAS was used due to its simplicity and due to its acceptability as a generic pain measure. We asked for the highest measure of pain within the first 12 hours, on POD1 and on POD2.

### 2.5. Sample Size Estimate

A total of 60 patients were intended to be enrolled in the study. However, the departure of the primary orthopedic joint replacement surgeon caused our sample size to be reduced significantly.

### 2.6. Data Analysis

The data compiled blindly and anonymously by the study team was transferred into a confidential study database. Data was analyzed as intent-to-treat. The investigators, with the assistance of the statistician, conducted an analysis using a two-sample Kolmogorov-Smirnov test, a two-tailed test. The primary endpoint is quality of sleep as evidenced by the modified PSQI questionnaire.

## 3. Results

### 3.1. Recruitment and Retention of Patients

Patients were recruited between years 2010 and 2011. The patients screened were undergoing either a total knee arthroplasty (TKA), total hip arthroplasty, or a hip fracture repair. A total of 36 additional patients were screened. The three most common reasons why patients were not enrolled included the following: (1) patient did not speak the English language, (2) patient had obstructive sleep apnea, and (3) patients were having a revision surgery. A total of 29 patients were used for the study.

### 3.2. Baseline Characteristics and Clinical Variables

The placebo and gabapentin subject groups were comparable in terms of age, sex, and ASA status (Table 1).

### 3.3. Primary Outcome

Outcomes in the pre-op PSQI questionnaire and the post-op sleep questionnaire for POD1 and POD2 for the placebo and gabapentin groups can be found in Tables 2(a) and 2(b), respectively. As expected, there was no significant difference between the PSQI scores of the placebo and gabapentin groups the morning prior to the surgery. Difference in sleep quality between the two groups on POD1 was also found to be statistically insignificant. Although sleep quality did seem to improve in POD2 for the gabapentin group, the *P* value was found to be 0.64. Thus, no significant differences were found on these questionnaires.

### 3.4. Secondary Outcomes

As previously mentioned, we used the VAS as our secondary outcome due to its simplicity and due to its acceptability as a generic pain measure. We asked for the highest measure of pain within the first 12 hours, on POD1 and on POD2. There was no significant difference in pain scores between the gabapentin and placebo groups within the first 12 hours after surgery. This result was expected, due to the effects of the peripheral blocks performed perioperatively. We also found no significant difference in pain scores between the gabapentin and placebo groups on both POD1 and POD2.

## 4. Discussion

This study was designed with the aim of examining whether gabapentin in the context of peripheral nerve blocks and opioid PCA would show an improvement in sleep quality. Though no specific dosage of gabapentin has been shown to be optimal, we selected to use a 400 mg preoperative dose. The present study did not find a significant difference between gabapentin and placebo groups in terms of the self-reported sleep quality questionnaire on either POD1 or POD2. Furthermore, pain scores on POD 1 and POD 2 did not differ between groups. Although the aforementioned studies and meta-analyses have shown a positive correlation between gabapentin use and reduction of postsurgical pain, it is clear that more evidence is needed to determine whether gabapentin has a positive effect on sleep quality. The results obtained from this study are unable to support the hypothesis of improved sleep quality in patients who receive gabapentin perioperatively, as opposed to patients who do not receive this adjunctive treatment.

With regard to pain scores, immediate postoperative pain was minimal equally amongst both groups, which is expected with effective sciatic and femoral nerve blocks. However, even as local anesthetic effect dissipated with time, there still appeared to be no difference in pain scores on POD1 and POD2 between patients who received gabapentin and those allocated to the placebo group. The results of this study should not be generalized to alternative perioperative pain regimens (i.e., regimens without a peripheral block or opioid PCA) as these results were specific to our regimen. Furthermore, the power of this study, which was expected to be greater when the study was first designed, was greatly compromised due to the unexpected departure of the orthopedic surgeon performing these operations. In conclusion, a single preoperative dose of gabapentin 400 mg followed by a second dose of gabapentin 400 mg on POD1 showed no significant improvement on self-reported sleep quality or pain scores on POD1 and POD2. Future studies should consider using the same standardized version of the PSQI preoperatively and on POD1 and POD2 in order to allow for more uniform comparison between different studies.

## Figures and Tables

**Table 1:** Demographics of patients in the control (placebo) versus study group (gabapentin).

	Gabapentin (*n* = 17)	Placebo (*n* = 12)
Mean patient age (yr)	54.6	53.33
Sex (Male/Female)	7/10	6/6
ASA		
Class I	1	0
Class II	5	5
Class III	11	7

**(a):** 

Placebo statistic	PSQI	Quality of sleep POD1 (Scale 0–3)	Quality of sleep POD2 (Scale 0–3)
(*n*)	12	12	12
Mean	7.333	1.333	1.000
Variance (*n* − 1)	18.970	0.424	0.727
Standard deviation (*n* − 1)	4.355	0.651	0.853

**(b):** 

Gabapentin statistic	PSQI	Quality of sleep POD1 (Scale 0–3)	Quality of sleep POD2 (Scale 0–3)
(*n*)	17	17	17
Mean	6.765	1.235	0.647
Variance (*n* − 1)	12.691	1.191	0.743
Standard deviation (*n* − 1)	3.562	1.091	0.862
